# Marine Bioprospecting, Biocatalysis and Process Development

**DOI:** 10.3390/microorganisms10101965

**Published:** 2022-10-05

**Authors:** Carlos J. C. Rodrigues, Carla C. C. R. de Carvalho

**Affiliations:** 1 Department of Bioengineering, iBB—Institute for Bioengineering and Biosciences, Instituto Superior Técnico, Universidade de Lisboa, Av. Rovisco Pais, 1049-001 Lisbon, Portugal; 2 Associate Laboratory i4HB—Institute for Health and Bioeconomy, Instituto Superior Técnico, Universidade de Lisboa, Av. Rovisco Pais, 1049-001 Lisbon, Portugal

**Keywords:** cultivation, metagenomics, biocatalyst, bioprocess, marine biotechnology

## Abstract

Oceans possess tremendous diversity in microbial life. The enzymatic machinery that marine bacteria present is the result of extensive evolution to assist cell survival under the harsh and continuously changing conditions found in the marine environment. Several bacterial cells and enzymes are already used at an industrial scale, but novel biocatalysts are still needed for sustainable industrial applications, with benefits for both public health and the environment. Metagenomic techniques have enabled the discovery of novel biocatalysts, biosynthetic pathways, and microbial identification without their cultivation. However, a key stage for application of novel biocatalysts is the need for rapid evaluation of the feasibility of the bioprocess. Cultivation of not-yet-cultured bacteria is challenging and requires new methodologies to enable growth of the bacteria present in collected environmental samples, but, once a bacterium is isolated, its enzyme activities are easily measured. High-throughput screening techniques have also been used successfully, and innovative in vitro screening platforms to rapidly identify relevant enzymatic activities continue to improve. Small-scale approaches and process integration could improve the study and development of new bioprocesses to produce commercially interesting products. In this work, the latest studies related to (i) the growth of marine bacteria under laboratorial conditions, (ii) screening techniques for bioprospecting, and (iii) bioprocess development using microreactors and miniaturized systems are reviewed and discussed.

## 1. Introduction

Microorganisms are the oldest form of life on Earth [1]. These organisms evolved over millions of years and colonized virtually every habitat. Their adaptation to different environmental conditions made them the most diverse organisms, either taxonomically, metabolically, or functionally [2]. It has been predicted that Earth hosts one trillion species (10^12^) of microorganisms, of which only 0.001% have been discovered so far [2]. Marine environments are interesting places for bioprospecting new microbial species with putative commercial application. These underexplored habitats are characterized by a wide range of abiotic conditions that lead to rich biological and genetic diversity [3]. The challenging conditions of some of these places made microorganisms develop specialized enzymes and metabolites with unique biochemical characteristics [4].

Assessment of the tremendous biodiversity of marine environments has been carried out using both culture-dependent and -independent techniques. During the “golden age” of bacteriology, Robert Koch, Fannie Hesse, Julius R. Petri, and other scientists, developed cultivation techniques in solid media [5] that enabled isolation of microorganisms in pure cultures. Marine medium containing nitrogen, vitamins, amino acids, and minerals necessary for growth of marine bacteria was developed by Claude E. Zobell in 1941 [6]. This medium enables cultivation of aerobic heterotrophic marine bacteria. However, several studies published throughout the 20th century led to the assumption that only a small proportion of the total bacteria in a sample could grow under laboratory conditions. Among them is the work of Razumov, in 1932, where the number of cells counted by microscopy in samples from oligo- and mesotrophic habitats was several orders of magnitude higher than that counted by the spread plate technique [7]. The “great plate count anomaly” was further supported by the work of Staley and Konopka in the 1980s, where it was stated that only 1% of the heterotrophic bacteria from oligotrophic to mesotrophic aquatic habitats could be recovered by cultivation [8,9]. This led to the currently common assumption that only 0.1–1% of microorganisms present in a marine sample may grow under laboratory conditions.

In 1990, one of the first studies using a culture-independent approach to access the diversity of bacterioplankton from the Sargasso Sea [10] opened new perspectives in microbiology since it showed the presence of novel microbial groups identified by rRNA gene sequencing without their culture. Further development of sequence technology resulted in so-called metagenomics, a molecular technique able to describe microbial populations from environmental samples [11]. During the first targeted metagenomic sequencing, conducted by Stein et al. in 1996, a genome fragment with 40 thousand base pairs from uncultivated marine archaea was obtained from the marine picoplankton assemblage by PCR amplification of the 16S rRNA gene [12]. The use of high-throughput DNA sequencing with recent bioinformatic tools has enabled the discovery of enzymes by sequence-based approaches targeted at gene families, and function-screening strategies for the discovery of novel gene sequences with desired functions [13].

Regardless of the significant advantages, metagenomic studies also show some weaknesses, including: cloning and sampling bias; incorrect promoter sites; 16S rRNA chimeras and artificial replicates leading to inaccurate estimation of microbial diversity; and inaccurate reconstruction of metabolic pathways [14,15,16,17]. This has led, in recent years, to several scientists referring to the importance of still cultivating microorganisms under laboratory conditions while showing that it is probably the most valuable approach to studying environmental communities and microbiomes and to finding new biocatalytic activities and secondary metabolites [18,19,20,21]. Nevertheless, growth of marine microorganisms in the laboratory is considered to be very difficult, with most studies using metagenomics stating that only 0.1–1% of marine bacteria may grow in the laboratory. The difficulty to mimic natural conditions in vitro and the lack of time to test numerous media formulations necessary for the growth of “unculturable” bacteria are some of the reasons for the perpetuation of the dogma that the majority of environmental bacteria do not grow in the laboratory [22,23]. However, recent studies have showed that a much larger percentage of marine bacteria may be isolated [24,25], including one study from our laboratory comparing cultivation-dependent and -independent techniques [24], and a study using a continuous-flow down-flow hanging sponge bioreactor to favor growth of deep sediment microorganisms [26].

The need to manufacture products for the increasingly growing world population using sustainable industrial processes further favors the interest in biocatalytical applications at the industrial level [27,28,29]. Studies employing new classes of enzymes, non-natural reactions, and metabolic engineering are paving the way to new industrial applications [30]. Marine biocatalysts, either as whole cells or their enzymes, may operate under mild and environmentally friendly conditions, similar to their terrestrial counterparts. However, their well-recognized habitat-related features, such as salt tolerance, thermostability (e.g., to high temperatures observed in extremophiles collected in hydrothermal vents; to cold temperatures in extremophiles isolated from deep sea), and barophilicity, make them desirable for new industrial sustainable process applications [4,25,31,32]. However, implementation of a biocatalytic bioprocess at a large scale requires rapid data acquisition to assess conditions and evaluation of the process at low cost [33]. Miniaturized bioreactors have been successfully used to address the challenge in the early stages of process development to solve scale-up problems and, at the same time, reduce costs [34,35].

In the present manuscript, the new developments in marine bioprospecting for finding efficient biocatalysts by culture-dependent techniques and for assessing the conditions enabling their use for the production of valuable compounds is discussed. A review of marine biocatalysts found to be able to improve industrial processes and how such bioprocesses may be developed is also presented. 

## 2. Marine Biocatalysts to Improve Industrial Processes

The global enzyme market value is forecasted to reach USD 14,507.6 million in 2027, with a mean annual growth rate of 6.5% from 2020 to 2027 [36]. Currently, the most commonly used enzymes are proteases, cellulases, lipases, hydrolases, polymerases, amylases, esterases, xylanases, and transaminases. They are used in processes across the food processing, pulp and paper, textile and leather, chemical, and pharmaceutical industries [28,37,38,39,40].

The metabolic function and biomolecular machinery of marine biocatalysts are influenced by the diverse conditions that the oceans encompass regarding, e.g., pressure, temperature, salinity, and solar exposure [3,4]. Enzymatic properties, such as halo- and thermostability, of marine biocatalysts could be extremely interesting for industrial applications. In bioprocesses using high salt concentrations, these cells or their enzymes may be particularly interesting. Halophilic enzymes maintain activity and stability under low water activity conditions due to important factors, such as increased ion-pair networks, reduced hydrophobic surface patches, and a high number of ordered side chains [41,42]. This property makes halophilic enzymes suitable to work in the presence of organic solvents [43,44] and particularly interesting to the industry [45]. For example, a halophilic α-amylase from *Nesterenkonia* sp. strain F presented a half-life in the absence of an organic solvent of ca. 54 days but presented a half-life longer than 79 days in the presence of 20% of organic solvents with log *P*_ow_ ≥ 1.97 [46]. Application of an amylase from the marine bacterium *Pseudoalteromonas undina* NKMB 0074 enabled the conversion of sugars for bioethanol production from microalgae biomass under saline conditions without a desalinization step, which would be necessary if amylases from terrestrial origin were to be used [47].

Enzymes stable in a wide range of pH values can also be found in marine environments and can be used, e.g., in the detergent industry [48,49]. Marine habitats, such as thermal vents, may also be a source of thermophilic biocatalysts with hyper thermostability properties (80–108 °C) and barophilicity [50,51]. These characteristics are important in reactions that require high temperature and/or pressure to occur. Adaptation of biocatalysts to low temperatures is also good for biotechnological exploitation: cold-adapted enzymes are needed for the detergent and food industries, or for bioremediation processes since they enable lower energy consumption, reduced risk of microbial contamination, and reduced instability of reactants and/or products observed at higher temperatures [31,52]. For example, the lipolytic capacity of Antarctic cold-adapted marine bacteria could be a source of exploitable enzymes [53].

Examples of enzymes from marine bacteria isolated in the last two decades by culture-dependent methods are shown in Table 1. A diverse range of enzymes with different operational conditions and potential applications have been described.

## 3. Assessing Not-Yet-Cultured Biocatalysts

Isolation and cultivation of microorganisms in laboratories have been studied since the 19th century. The use of the spread plate technique on solid agar, known today as a classical method, was essential for the initial discovery of microorganisms associated with diseases [5]. In the “molecular era”, the description of microbial diversity in environmental habitats was substituted by metagenomic studies. The use of high-throughput sequencing directly to a sample, from a complex ecosystem, revolutionized the way scientists looked to microbial diversity in nature [11,66]. Sampling, isolation, and analysis of DNA led to identification of novel genetic diversity that was not detected using the tedious classic task of growing microbes on agar plates [67,68]. Metagenomic techniques enabled scientists to discover microbial ‘dark matter’ that was not accessible by culture-dependent methods [69]. Big data and artificial intelligence technologies improved mining of microbial dark matter to study microbial communities and their biocatalytic potential [70,71], in some cases assisted by sophisticated proteomics and metabolomics analyses [72,73].

Most culture-independent techniques require creation of metagenomic libraries, where DNA fragments are inserted into small lambda phage vectors and plasmids or large cosmid or fosmid vectors depending on the target genes [74,75]. Then, phenotypic detection [76,77], heterologous complementation of host strains or mutants [78], and induced gene expression [79] are used to screen and produce new molecules/enzymes [80]. These approaches resulted in industrially relevant enzymes [76,80,81,82,83]. For instance, De Santi et al. discovered a novel cold-active and salt-tolerant esterase gene from marine sediments using functional screening to assess Arctic metagenomic libraries [32]. Li et al. discovered a novel *β*-galactosidase in a marine metagenomic library [84]. The enzyme revealed high hydrolytic activity and transglycosylation towards lactose. The results demonstrate the potential of this approach for the production of galacto-oligosaccharides to be used as prebiotics. Tchigvintsev et al. screened three marine metagenome gene libraries for esterase activity and found five highly active esterases, which were selected for biochemical characterization [85]. The esterases retained high activity at 5 °C, indicating that they were cold-adapted enzymes, hydrolyzed a broad range of monoester substrates, and several polyester substrates, such as polylactic acid. Ferrer et al. studied sequence and functional datasets of 288 α/β-hydrolase fold superfamily esterase–lipase enzymes from metagenomes and found that the majority of these enzymes came from soil (98 in total), but marine habitats provided the second most abundant environment (87 in total) for esterases–lipases [86].

Despite the potential of metagenomics, the methodology also has some limitations [14,87]. Expression of the specific gene sequence on a heterologous host is usually difficult, resulting in low observed targets, especially in *Escherichia coli*, where only 40% of enzymatic activities are detected by random cloning [88]. Additionally, the fact that about 7–60% of new sequenced genes have no attributed functions due to lack of database representatives creates gaps in the sequences, increasing the difficulty in predicting and detecting novel enzymes [89,90]. Other limitations include the difficulty to compare results between laboratories because of different DNA extraction and manipulation procedures, the fact that gene expression is not inputted in gene information, and that metabolic and biochemical diversity cannot be comprehensively described by sequence analysis alone [19,85]. This requires meta-transcriptomics, meta-proteomics, genome assembly, cultivation techniques, and activity/based screening methods, such as agar-plate-based screenings [80,91,92]. This latter approach has been successful to screen metagenomic libraries for, e.g., esterase, lipase, and cellulase activities [85,86,91].

Another key point is the increasingly demanding bioinformatic and computational resources, including computing power and computational skills, to analyze and exploit metagenomic sequences. In a recent study, Grealey et al. estimated that the carbon footprint of metagenome assembly with three commonly used assemblers (metaSPAdes, MEGAHIT, and MetaVelvet) ranges between 14 and 186 kg CO_2_e (kilograms of CO_2_ equivalent units), corresponding to 0.14 to 1.9 kg CO_2_e per sample [93].

### Techniques for Cultivation of Marine Bacteria

The efficiency of cultivating microorganisms from a diverse number of environments is low, although this does not mean that they are not culturable [23]. The dogma coined originally in 1985 by Staley and Konopka [8] was eventually generalized to all kinds of environmental samples. It is currently well accepted by the scientific community that only 1% of bacteria from the environment grow under laboratory conditions, although the ‘great plate count anomaly’ has been proven not to be true [24,94]. The dogma started because only a small part of the population visible under the microscope provided colonies on agar plates. However, the original study referred to growth of heterotrophic bacteria from samples collected in oligotrophic to mesotrophic aquatic habitats [8,9]. The perpetuation of the low percentage of bacteria being cultivable results from the inability of scientists to mimic in the laboratory the conditions (such as nutrient type and concentration, pH, osmotic conditions, and temperature) found in the locality where the cells were collected [18,22,94,95]. 

The 21st century assisted to the rebirth of culture-dependent strategies to explore microbial diversity [19,21,95,96,97,98]. Study of the human microbiota is an excellent example of the potential and success of high-throughput culture techniques [87,99]. Lagier et al. combined various selective and/or enrichment culture conditions coupled to mass spectrometry (MS) techniques with matrix-assisted laser desorption ionization time-of-flight (MADI-TOF) to study the human microbiome [100]. The approach resulted in 32,500 colonies, which included 174 species never described previously in the human gut, and 31 new species. Culturomics identified 17% more bacterial species in comparison with the metagenomic analysis of the same samples. Other studies of the human gut microbiome also noted the advantages of culturomics over metagenomics [101,102].

Several works have shown that significantly more than 1% of microbial species could be grown and that cultivation techniques (including the use of multiple culture media, refinement of culture media composition, and co-culture of multiple species) could detect isolates not detectable by biased metagenomic techniques [24,94,103]. We have recently shown that we could grow ca. 45% of the cells in a sample from a marine rock pond using marine agar medium after spread-plating the cells on different agar media compositions and concentrations, and by waiting for 6 weeks [24]. Although metataxonomy showed members of ten different phyla of which only four were isolated by the culture-dependent method, the latter detected four taxonomic orders not detected by the cultivation-independent technique. These include bacteria from the phyla Bacillota and Actinomycetota.

The nutrients present in habitats, such as ocean environments, could be very scarce [104]. Microorganisms growing in low-nutrient habitats are called oligotrophs [105]. They are adapted to grow in low-nutrient concentrations, and most of them may grow slowly or be unable to grow well in nutrient-rich habitats. On the other hand, copiotrophs are microorganisms that can only grow in high-nutrient concentration conditions and are not fitted to grow under low-nutrient conditions as oligotrophs and may even enter a dormant state [105,106,107]. Copiotrophs are more common in laboratory and industry applications because of their fast growth rates and easier culture maintenance.

**Figure 1:**
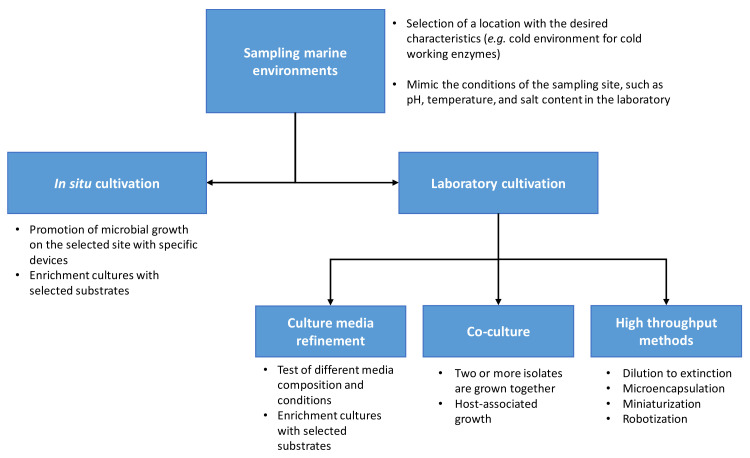
New cultivation approaches for isolation of microbial species from marine environments.

Classical microbiological methods, also known as standard spread plating, use standard cultivation media and reduced incubation times, resulting in growth of copiotrophic over oligotrophic bacteria. To prevent this, new methodologies to mimic natural conditions include the following strategies (Figure 1): modification and optimization of media composition with specific chemical(s); dilution of the media; adaptation of the incubation conditions (temperature, pH, salt concentration, etc.); and prolonged time of incubation, enabling growth of slow growing bacteria. For example, the work developed in our laboratory using different solid media compositions and concentrations to promote the growth of bacteria present in samples collected at the Azores (Portugal) enabled isolation of 205 strains of different genera [25]. Different media compositions were used, namely: tryptic soy agar (TSA), thioglycollate with resazurin (Thio), sea salts, sea salts with 5 g/L glucose, and mineral medium supplemented with 5 g/L glucose and 3.5 g/L of yeast extract. To promote the growth of oligotrophs, 10- and 100-fold dilutions of TSA and Thio media were used, resulting in growth of strains not observed in the regular concentration. A recent study by Jung et al. showed that, by using diffusion chambers, dilution-to-extinction culture, and modified agar preparations, 201 novel species that did not grow using the classical methods could be grown [108]. Other techniques that have been found to be successful include cultivation with water from the sampling site to better mimic the natural conditions [109], while microfluidics, next-generation 3D bioprinting, and single-cell metabolomics may revolutionize microbiology [110]. These improvements to the traditional techniques increase the chances of finding the best nutritional conditions necessary for bacterial growth.

Dilution-to-extinction techniques are used to obtain a low number of cells (5 to 10 cells) per well in microtiter plates through separation of the initial microbial community. The experiments could take several weeks, and the growth may be monitored by microscopy [111]. However, some bacterial cells are unable to grow in the laboratory unless they have the assistance of other(s) bacterium(a) (helper strain). This co-culture approach enables growth of a bacterium in a Petri dish because the helper strain produces compounds, such as amino acids, vitamins, fatty acids, reduced sulfur compounds, siderophores, and/or electron shuttles, necessary for its growth [112,113,114,115]. These compounds perform important functions on the metabolism and are essential to the growth of a given strain, which must acquire them from the neighbor producing cells. This demonstrates the importance of biotic interactions for microbial communities in the natural environment [104]. To study such interactions, some approaches bring the natural environment to the laboratory. For example, Kaeberlein et al. were able to grow bacteria using diffusion chambers in a simulated aquarium environment. The bacteria only grew in the presence of the other elements of the natural community [116]. 

New efforts to mimic the environmental conditions of yet-to-be-cultivated bacteria have been developed [23,97], which provided information not provided by metagenomics. For example, the reversible tricarboxylic acid (TCA) cycle of a marine bacterium isolated from a deep-sea hydrothermal field was discovered after cultivation [117]. The highly efficient and reversible citrate synthase that requires reduced ferredoxin was undetectable by metagenomic analysis. The results obtained contributed to the understanding of the origins of life on Earth.

Another important concept to take into consideration is the physiological state of cells. In their natural environment, cells could be viable but in a state of deep dormancy. It is believed that this state, where cells are viable but not culturable (VBNC), is related to unculturability of indigenous bacteria [118] and could be a characteristic of starvation–survival response [119]. The VBNC may comprise the yet-to-be-cultured cells [120]. For resuscitation of bacteria from such a state, efforts around the world are being carried out to mimic the various habitats colonized by microbial species and to develop new cultivation methods [95,121,122]. While abiotic factors, such as nutrients, osmotic pressure, pH, and temperature, are easily recognized, biotic factors necessary for cell interactions and “resuscitation” are largely unknown. Jung et al. used in situ cultivation with a diffusion chamber to isolate previously uncultivated microbial species living in the marine sponge *Theonella swinhoei* [123]. In total, 40% of the in situ isolates had not been previously grown. When the authors added sponge extract to agar plates, they found that the sponge extract contained a growth initiation factor that does not continuously promote growth activity but triggers regrowth of cells and is likely promoting resuscitation from dormancy state. Several compounds have been found able to promote the growth of dormant cells, including the resuscitation-promoting factor, siderophores, and pyruvate (for a review, see Ref. [124]).

Taking advantage of the strategies stated before, some methods use high-throughput cultivation approaches. A recent review details the innovations that were achieved to culture new microbes [125]. These include cultivation methods based in membrane diffusion, microfluidics, and cell sorting techniques. Membrane filters and diffusion chambers could be deposited in the natural habitat to promote growth of certain bacteria. These techniques maintain the cells separated from the community but enable exchange of important molecules for their growth. For instance, Steinert et al. implanted diffusion growth chambers in the tissue of *Rhabdastrella globostellata* reef sponges. After one month of incubation and posterior analysis, fifteen previously uncultivated marine bacteria were identified [126]. Using the same principles as described above, Nichols et al. developed a high-throughput cultivation technique using an isolation chip, which was called Ichip [127]. The Ichip has 384 through-holes of 1 mm of diameter that enable immobilization of cells up to a precision of one cell inside each of the small agar plugs. The results obtained with the incubation of the Ichip in marine and soil environments demonstrated that the number of colonies is higher than in diffusion chambers and Petri dish techniques. Moreover, only one species overlap was found by the authors between the species obtained with the Ichip and Petri dish techniques. 

These several cultivation techniques demonstrate the possibility to culture novel yet-to-be-cultivated bacteria. Combination of different techniques and understanding of the natural conditions and high-throughput approaches will contribute to demystifying the idea of “unculturable” bacteria.

## 4. Detection of Biocatalysts from Cultured Bacteria

Different strategies have been applied for screening of novel marine products and enzymes [32,80,108]. Taking into consideration the importance of culture-dependent techniques discussed before, only screening strategies dependent on cultivation of microorganisms and libraries containing natural isolates will be introduced and discussed in the present review.

**Figure 2:**
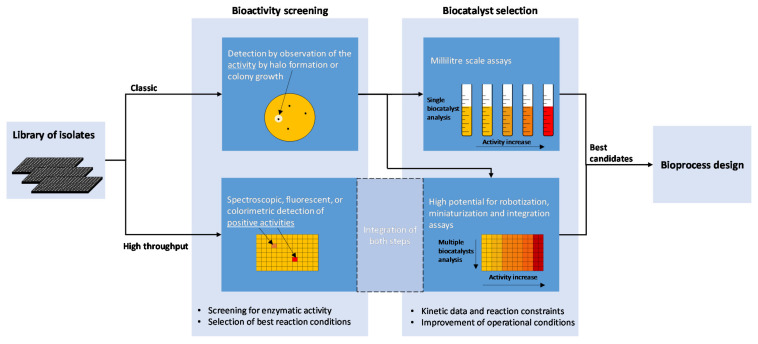
Traditional and high-throughput approaches for screening and selection of biocatalysts from a library of isolates, and assessment of the best bioreaction conditions.

Conventional methods to find biocatalysts are based in activity screening, with Petri-dish-based techniques being the most used methods for both cultivated isolates and for screening of metagenomic gene libraries (Figure 2). The Petri dish medium is prepared with the substrate for which the desired enzyme should be active. Identification of positive results is made by the formation of colonies able to grow on the substrate and/or changes in the media, such as formation of halos or color changes surrounding the colonies. De Santi et al. screened different enzyme activities in 100 marine isolates from Artic islands [32]. Marine agar plates supplemented with different substrates for each enzyme type were used, including tributyrin, skim milk, chitin, carboxymethylcellulose, gelatin, starch, xylan, and DNA. The bacterial cultures were spotted onto the assay plates, which were incubated at 4 and 20 °C. After 1 week, protease, esterase/lipase, chitinase, cellulase, gelatinase, amylase, xylanase, and DNase activities were detected. This study identified interesting cold-adapted enzymes for potential industrial applications.

In our laboratory, 27 *Bacillus* sp. isolated from samples collected in the Azores (Portugal) were screened for the presence of proteases, inulinases, amylases, lipases/esterases, and cellulases [25]. Agar plates with medium containing the substrates for the sought enzymes were inoculated with the marine *Bacillus* isolates and incubated at 30 and 37 °C for 48 h. Several *Bacillus* strains were able to grow on the different agar plates. Among them, four *Bacillus* isolates showed significant activity towards the substrate inulin and were selected for further studies. The highest activity was achieved with an isolate identified as *B. subtilis*, corresponding to 2.2 g sugars/g_protein_.h, in a magnetically stirred bioreactor.

Although these conventional screening methods for discovery of novel biocatalysts are still used successfully, high-throughput methods are needed for screening of libraries containing natural isolates and metagenomic clones (Figure 2). High-throughput colorimetric screening assays are usually developed, enabling rapid assessment of the conversion of wanted substrates. A high-throughput colorimetric screening assay based on pH changes, capable of detecting and distinguishing nitrilases and nitrile hydratases, was reported by Angelini et al. [128]. From the 130 isolates tested, twelve were nitralase producers, three were NHase-amidase producers, and five isolates produced both enzymes. Another colorimetric method, based on the formation of a red precipitate, was developed for screening of transaminase activity by Baud et al. [129]. The method could be used to test different ketones/aldehydes as substrates for transaminase activity in clarified cell lysates both in liquid phase in multi-well plates and in solid phase as a colony-based colorimetric assay for rapid screening of transaminase activity in mutant libraries.

A method using the same colorimetric reaction but integrating screening and determination of biocatalyst activity in real time was developed in our laboratory [130]. The method uses image analysis for quantification of the color of the reaction mixture, which changes according to the transaminase activity of the wild isolates being tested. The developed method reduces the time required for detection and quantification of specific transaminase activity while reducing costs since expensive chromatographic analyses are not necessary. The integrated method enables rapid screening and evaluation of isolate libraries, thus helping to select potential biocatalysts for the next step of bioprocess development.

## 5. Bioprocess Development

**Figure 3:**
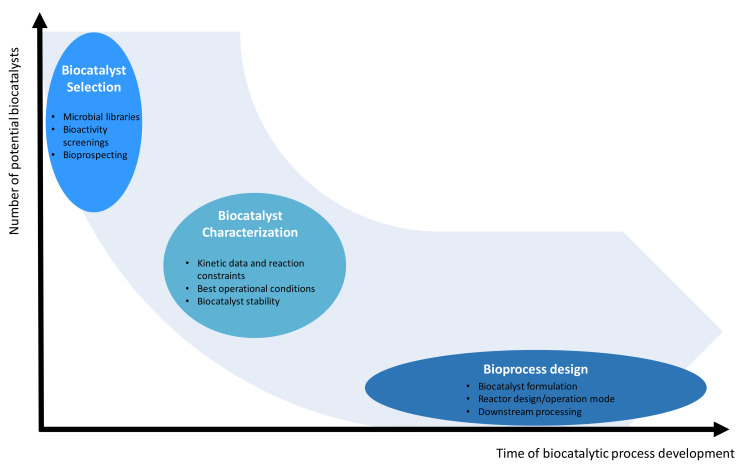
Conceptual visualization of the simultaneous number of biocatalysts to be tested during the different steps of the biocatalytic process development. At the early stages, a large number of putative candidates are tested, preferably by using high-throughput assays. After identification of the most active candidates, the effect of reaction conditions on kinetic data and biocatalyst stability are tested. The best candidate is then selected for the development of the production process, where biocatalyst formulation, reactor type and operation mode, and extraction and purification of product(s) are tested and defined.

The increasing demand for new biocatalysts, and their application in industrial processes to assist conventional chemical processes, should be connected to bioprocess development [131,132]. During development of a biocatalytic process, the following steps should be considered: (i) biocatalyst selection, (ii) biocatalyst characterization/improvement, and (iii) bioprocess design, including product recovery and purification. The number of putative biocatalysts being simultaneously studied decreases during the time spent on development of the biocatalytic process as several candidates are eliminated (Figure 3). On the other hand, the time required for each step increases since characterization of the biocatalyst and downstream processing are time-consuming. 

Application of biocatalysts in industrial processes requires in-depth investigation and full understanding of the process. Therefore, relevant data have to be acquired for successful development and implementation of a bioprocess on a large scale. The challenge resides in fast acquisition of reliable data while at the same time reducing costs [27]. For that, low volume approaches using micro- and small reactors, microtiter plates, microfluidic devices, and shake-flasks have been used in the early stages of process development to obtain a large array of process data at reduced costs when compared to bench-scale bioreactors [35,133,134,135,136]. The low volume of microreactors and microfluidic devices enable the use of low amounts of reagents and biocatalyst(s) while enabling good control of biocatalytic reaction variables [33,34,136,137]. Furthermore, microsystems are easily integrated with sensor technology, enabling faster bioprocess development [138,139,140]. The last step of bioprocess design needs to ensure that every variable in the process is optimized and ready for implementation.

We studied in our laboratory the production of benzyl alcohol by *Glutamicibacter arilaitensis* 232 isolated from a marine sample [141]. The biocatalyst was identified during screening using ketones and aldehydes as substrates. The need for a cosolvent to help the solubilization of the substrate benzaldehyde, the effect of the addition of an organic solvent to act as a substrate reservoir, and the effect of substrate concentration on biocatalyst activity and inhibition were determined in 1.5–10 mL bioreactors. The data collected were used for the scale-up of the system to a 100 mL fed-batch stirred reactor and to a 25 mL continuous plug flow reactor (CPFR) using both free and immobilized cells. The two-phase stirred reactor system enabled a productivity of 0.122 g_benzyl alcohol_/g_cell dry weight_.L.h, but benzyl alcohol concentrations above 50 mM had a strong inhibitory effect on free cells. The CPFR with immobilized cells was efficient in surpassing product inhibition, and the maximum achieved productivity was 1.16 g_benzyl alcohol_/g_DCW_Lh.

In another study carried out by us, an ω-transaminase from a marine bacterium was investigated for bioconversion of bulky ketones to chiral amines [142]. The bioprocess development allowed the fast assessment of suitable reaction conditions. Inhibition of the amine donor for concentrations above 75 mM was observed. When five organic solvents were used as cosolvents for the amine acceptor, using mL-volume scale reactions, it was found that 25–30% (*v*/*v*) of dimethyl sulfoxide (DMSO) promoted the highest enzyme activity. Implementation of a two-phase system, with *n*-hexadecane as the organic phase, resulted in an increase in production of 15.9% in comparison with the single-phase aqueous system. The bioprocess studied has potential as an application for production of a building block for new pharmaceutical drugs.

The bacterium *Bacillus carboniphilus*, which was isolated in India, produced a cellulase able to carry out saccharification of pretreated rice straw, yielding ca. 15.56 g/L of reducing sugars at 96 h [143].

Vázquez et al. produced *Pseudomonas fluorescens* and *Phaeobacter* sp., which have been found to be probiotic bacteria for fish, being effective against fish pathogens and in helping larvae survival in several species [144]. The authors found that, by using peptones obtained from enzyme hydrolysates of fish discards, filtered seawater, and a low concentration of yeast extract, it was possible to decrease the cost of production of probiotic bacteria by 120 times.

## 6. Final Remarks

Marine habitats are an excellent source of novel biocatalysts and secondary metabolites that require more exploration. They are characterized by a wide range of conditions, which lead to rich biological and genetic diversity. Marine biocatalysts can perform remarkable and unusual bioprocesses because of their habitat-related features. The industrial interest in these properties is increasing, and more efforts to find suitable and efficient marine biocatalysts are being made.

Scientists are also focusing more on efforts to develop capable techniques for microbial cultivation in the laboratory. Understanding and mimicking the natural habitat parameters of the original microbes is essential. As discussed, the concept that microbes are unculturable under laboratorial conditions is outdated. The search for new biocatalysts should integrate both culture-dependent and -independent methods because distinct microorganisms may be identified by different techniques. New and better methods for culturing and screening of new biocatalysts are needed for the increasing demand of greener industrial bioprocesses. The marine environment is available and could sustain the market demand for novel biocatalytic properties. For that, techniques for rapid evaluation of activities and bioprocess efficiency are needed. Small-scale reactors are useful tools to improve bioprocess development at reduced costs and implementation at the industrial level. Overall, marine biocatalysts should contribute to sound sustainable industrial bioprocesses and to production of novel products necessary in diverse human activities.

## Figures and Tables

**Table 1:** Examples of enzymes from marine bacteria reported in the last two decades and their potential applications.

Enzyme	Marine Bacteria	Sampling Location and/or Organism	Isolation Technique	Enzyme Screening Technique	Activity	Optimal Temperature and pH	Potential Application	Ref.
Alkaline phosphatases	*Cobetia marina*	Mussels from Troitza Bay, Japanese Sea	Liquid culture	Standard assay for AP activity	Conversion of *p*-nitrophenylphosphate	50 °C; pH 10.3	Functional studies of nucleic acids and immunologic assays	[54]
Alkaline protease	Marine bacterium SD11	Sea mud, China	Agar plate	Bioactivity screening with agar plate	Hydrolysis of carbohydrates	60 °C; pH 10	Synthesis of peptides and detergent formulations in alkaline environments	[55]
α-Amylase	*Pseudoalteromonas* sp. M175	Antarctic sea ice	Flask culture	Genetic techniques	Cleavage of α-1,4-glycosidic linkages in starch molecules	25 °C; pH 8.0	Detergent additive to improve stain removal efficiency	[56]
κ-Carrageenase	*Pseudoalteromonas porphyrae*	Decayed seaweed collected from Yellow Sea, China	Medium containing carrageenan	κ-carrageenase activity	κ-Carrageenan hydrolysis	55 °C; pH 8.0	Production of sulfated oligosaccharides	[57]
Chitinase	*Moritella marina*	Sample from a depth of 1200 m, northern Pacific Ocean	Marine agar/broth containing colloidal chitin	Standard chitinase activity	*p*-nitrophenyl-β-1,4-*N*,*N*′-diacetyl-chitobiose	28 °C; pH 5.0	Waste management and biocontrol of pathogenic fungi	[58]
Dye-decolorizing peroxidase	*Actinobacteria* strain collection	Shallow water sediments, Trondheim fjord, Norway	Agar plate	Genomic data mining	Oxidization of several phenolic substrates	25 °C; pH 3–4	Degradation of natural or artificial dyes, or in chemical synthesis phenolic monomers	[59]
Esterase	*Thalassospira sp.* GB04J01	Sea fan, northern Norway	Flask culture	Genetic techniques	Hydrolysis of simple esters	45 °C; pH 8.5	Additives in laundry detergents	[60]
Fibrinolytic enzymes	*Bacillus subtilis* ICTF-1	Western seacoast of Maharashtra, India	Agar plate	Activity screening in microplate	Conversion of *N*-succinyl-Ala-Ala-Pro-Phe-*p*-nitroanilide	50 °C; pH 9.0	Drugs for the treatment of thrombosis	[61]
Inulinase (whole-cell)	*Bacillus subtilis*	Intertidal shallow water thermal vent, Azores	Agar plate	Bioactivity screening with agar plate	Hydrolysis of inulin to reducing sugars	40 °C; pH 8.5	Food industry	[25]
Lactase	*Pseudoalteromonas* sp. KNOUC808	Antarctic polar sea	Enrichments cultures	Hydrolysis of ONPG	Hydrolysis of o-nitrophenyl β-D-galactopyranoside	20 °C; pH 7.8	Detergent formulations, dairy industry, biosensors	[62]
Lipase	*Pseudomonas* sp. LSK25	Signy Island, Antarctica	Growth in LB broth at 4 °C	Tributyrin agar plates	Colorimetric assay with olive oil as substrate	10 °C; pH 7.0	Detergent, textile, and food industries	[63]
Sulfatase	*Pseudoalteromonas* sp.	Sea cucumber gut, Vietnam	Agar plate	Bioactivity screening with agar plate	Hydrolytic cleavage of sulfate ester groups on carbohydrates	68 °C; pH 6.5	Production of partially desulphated fucoidan oligosaccharides	[64]
Xylose isomerase	*Fulvimarina pelagi*	Sargasso Sea	Dilution-to-extinction	Genetic techniques	Interconversion of d-xylose and d-xylulose	35 °C; pH 7	Biofuels production	[65]

## Data Availability

Not applicable.
